# Supplementary material for: Nanoparticle-enhanced radiotherapy synergizes with PD-L1 blockade to limit post-surgical cancer recurrence and metastasis
Source: Nat Commun. 2022 May 20;13:2834. doi: 10.1038/s41467-022-30543-w (PMC9123179; doi:10.1038/s41467-022-30543-w)
Supplement: Supplementary file 1 — Supplementary Information [file 41467_2022_30543_MOESM1_ESM.pdf]

Supplementary Information for

Nanoparticle-enhanced radiotherapy synergizes with PD-L1 blockade to limit post-surgical cancer recurrence and metastasis

Xin Guan[1,2,3#], Liping Sun[1,2,3#], Yuting Shen[1,2,3#], Fengshan Jin[1,2,3], Xiaowan Bo[1,2,3], Chunyan Zhu[1,2,3], Xiaoxia Han[1,2,3], Xiaolong Li[1,2,3], Yu Chen[4*], Huixiong Xu [1,2,3,5*], Wenwen Yue[1,2,3*]

[1] Department of Medical Ultrasound and Center of Minimally Invasive Treatment for Tumor, Shanghai Tenth People's Hospital, School of Medicine, Tongji University, Shanghai, 200072, P.R. China.

[2] Ultrasound Research and Education Institute, Clinical Research Center for Interventional Medicine, School of Medicine, Tongji University, Shanghai, 200072, P.R. China.

[3] Shanghai Engineering Research Center of Ultrasound Diagnosis and Treatment; National Clinical Research Center for Interventional Medicine, Shanghai, 200072, P. R. China.

[4] Materdicine Lab, School of Life Sciences, Shanghai University, Shanghai, 200444, P. R. China.

[5] Department of Ultrasound, Zhongshan Hospital, Fudan University, 200032, P. R. China.

#These authors contributed equally.

*Corresponding author: E-mail: yuewen0902@tongji.edu.cn; xuhuixiong2022@126.com; chenyuedu@shu.edu.cn.

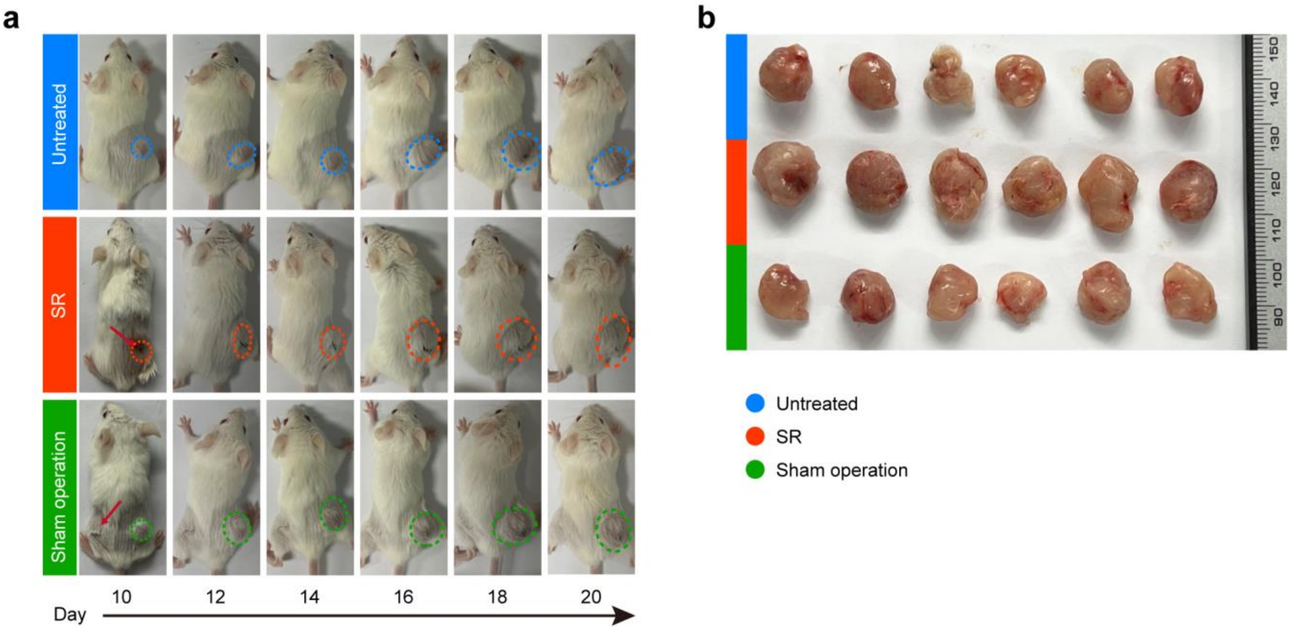

Supplementary Figure 1. SR accelerates local tumor progression. a Representative digital photos of the treated mice of six biologically independent animals from each group. The arrow indicates the surgical site, and the circle represents the tumor site. **b** Digital photos of the excised tumors on day 20 after varied treatments (n = 6 mice). SR, surgical resection.

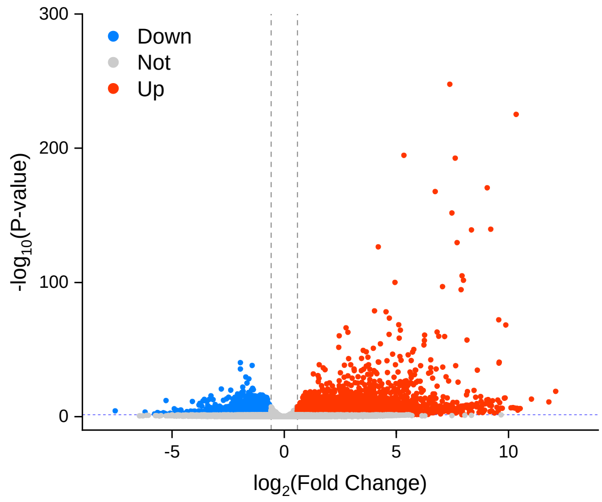

Supplementary Figure 2. Gene changes in transcriptome sequencing. Volcano map showing differentially expressed genes between untreated and SR-treated group. Differential expression analysis was performed using the DESeq2 (http://bioconductor.org/packages/release/bioc/html/ -DESeq2.html), DEGs with $\log_2$(foldchange) > 1.5 and Padj < 0.05 were considered to be significantly different expressed genes (n = 3 biologically independent samples). SR, surgical resection. Padj, P value adjusted by multiple hypothesis test.

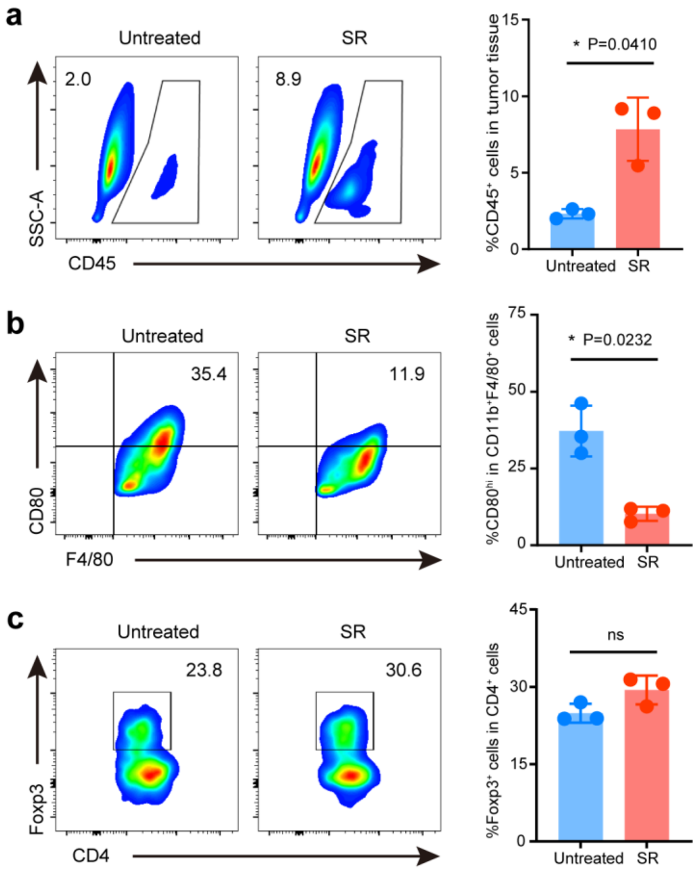

Supplementary Figure 3. SR affects the infiltration of immune cells. a-c Representative flow cytometric images and relative quantification of $CD45^+$ cells (**a**), TAM-M1 cells ($CD80^{hi}CD11b^+F4/80^+CD45^+$) (**b**), and Treg cells ($Foxp3^+CD4^+CD3^+CD45^+$) (**c**) within tumor tissues three days after SR treatment. SR, surgical resection. Data were expressed as means ± SD (n = 3 biologically independent samples in **a**, **b** and **c**). Statistical difference was calculated using two-tailed unpaired student's *t*-test. ns presents not significant, *P<0.05.

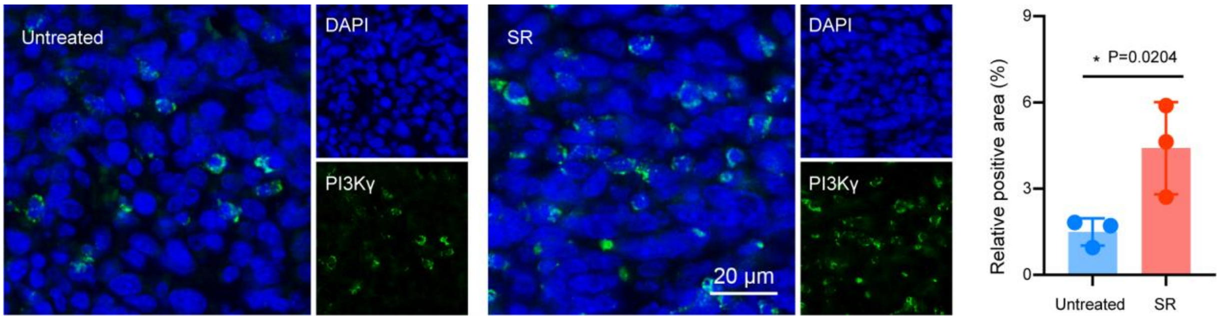

Supplementary Figure 4. PI3Kγ expression within tumor site. Representative immunofluorescence images and relative quantification of tumor slices stained with PI3Kγ antibody three days after SR treatment. Data were expressed as means ± SD (n = 3 biologically independent samples). Statistical difference was calculated using two-tailed unpaired student's *t*-test. *P<0.05. SR, surgical resection.

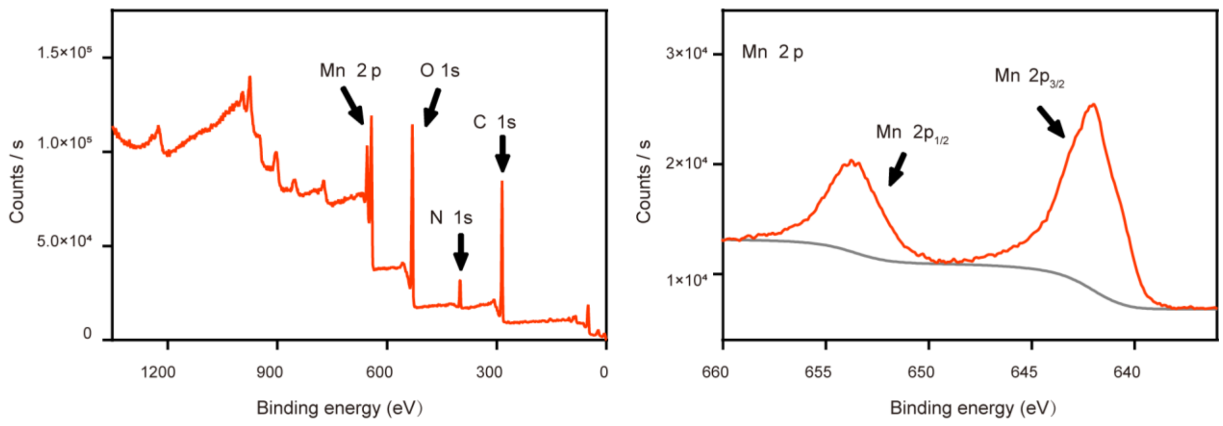

Supplementary Figure 5. X-ray photoelectron spectroscopy (XPS) characterization of HMP. Representative XPS full scan spectra and magnified spectra in the *Mn 2p* region of HMP from three independent experiments.

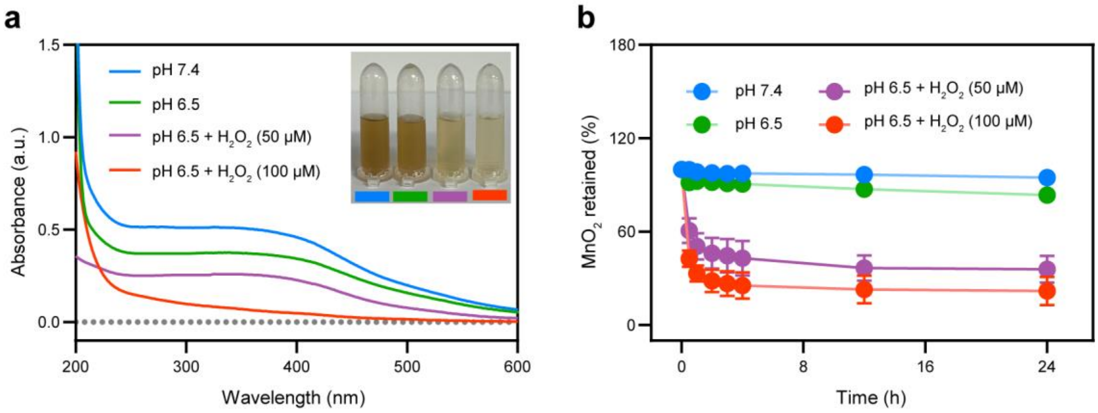

Supplementary Figure 6. Degradation behaviors of HMP. a Representative UV-vis spectrum and corresponding digital photos of HMP aqueous solution from three independent samples after reaction with varied media as indicated. **b** Accumulated degradation profiles of HMP dispersed in PBS (pH 7.4), PBS (pH 6.5) and PBS (pH 6.5) containing H_2O_2 (50 μM or 100 μM). Data were denoted as the mean ± SD (n = 3 independent samples).

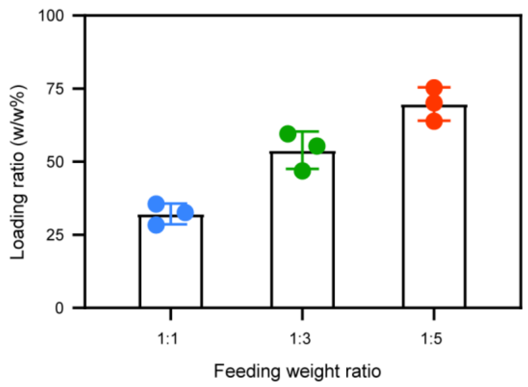

Supplementary Figure 7. Drug loading characteristics of HMP. Loading ratios of IPI549 in HMP at varied feeding doses determined by UV-vis spectrum. Data were denoted as the mean ± SD (n = 3 independent samples).

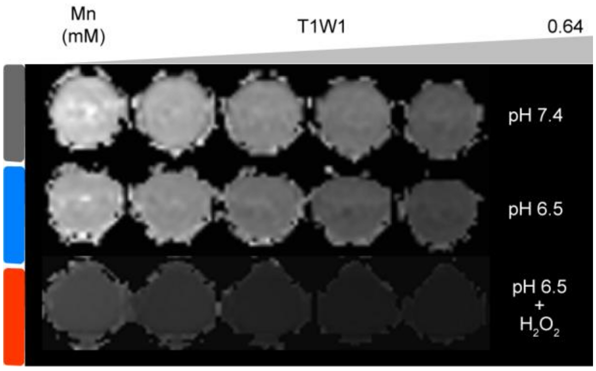

Supplementary Figure 8. Magnetic resonance imaging capacity of IPI549@HMP. Representative T_1-maps of different concentrations of IPI549@HMP dispersed in PBS (pH 7.4), PBS (pH 6.5) and PBS (pH 6.5) containing H_2O_2 (100 μM) from three independent samples. The experiments were repeated three times.

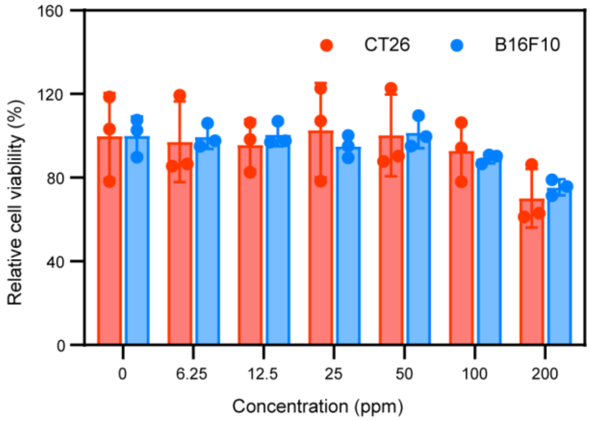

Supplementary Figure 9. *In vitro* safety evaluation of IPI549@HMP. Cell cytotoxicity of IPI549@HMP after co-incubation with CT26 and B16F10 cells for 24 h. The data were denoted as the mean ± SD (n = 3 independent experiments).

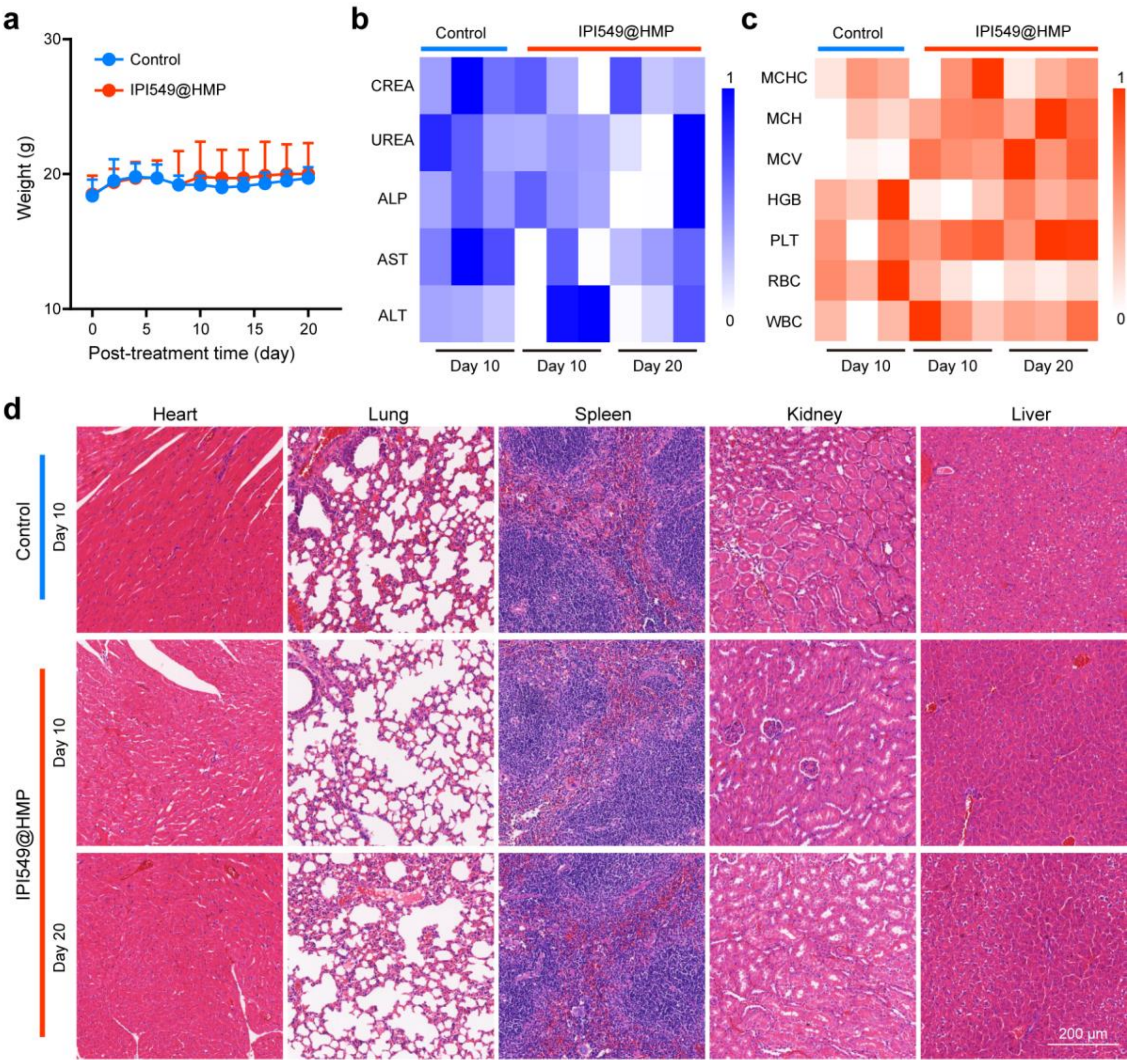

Supplementary Figure 10. *In vivo* safety evaluation of IPI549@HMP. **a** Body weight changes of BALB/c mice after injection of PBS or IPI549@HMP (dose of MnO_2 = 7.5 mg kg^{-1} and IPI549 = 1.5 mg kg^{-1}). Data were expressed as mean ± SD (n = 3 biologically independent animals). **b** Serum biochemistry data including creatinine (CREA), carbamide (UREA), alkaline phosphatase (ALP), aspartate transaminase (AST), alanine aminotransferase (ALT) were measured. Data were expressed as mean ± SD (n = 3 biologically independent animals). **c** Blood routine indexes including mean corpuscular hemoglobin concentration (MCHC), mean corpuscular hemoglobin (MCH), mean corpuscular volume (MCV), hemoglobin (HGB), platelet (PLT), red blood cell (RBC) and leukocyte (WBC) were measured. Data were expressed as mean ± SD (n = 3 biologically independent animals). **d** Representative H&E stained tissue sections of three biologically independent animals from each group (All scale bar = 200 μm)

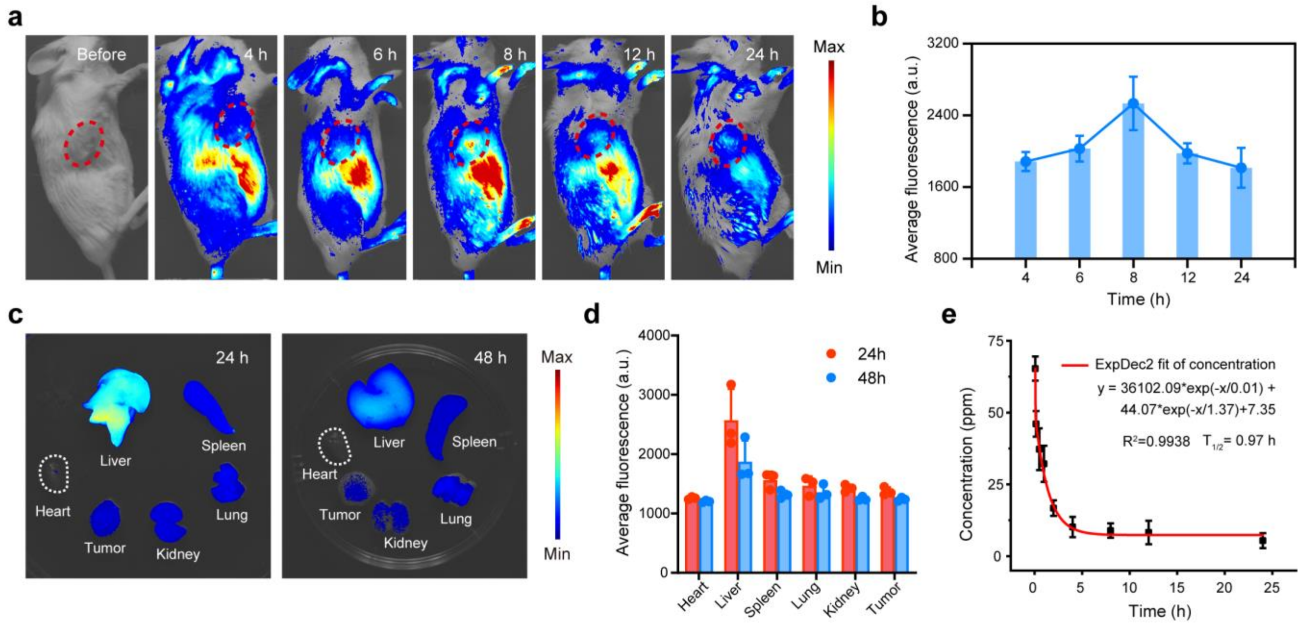

Supplementary Figure 11. ***In vivo*** **biodistribution and pharmacokinetics evaluation of IPI549@HMP**. **a**, **b** *In vivo* fluorescence images and relative quantification of CT26 tumor-bearing mice taken at pre-set time points post *i.v.* injection of IPI549@HMP. **c**, **d** *Ex vivo* fluorescence images and relative quantification of major organs and tumor dissected from mice at 24 h and 48 h. **e** Blood circulation curve of Mn concentration after *i.v.* administration of IPI549@HMP. A representative image of three biologically independent samples is shown in **a** and **c**. Data were expressed as mean ± SD (n = 3 biologically independent samples in **b**, **d** and **e**).

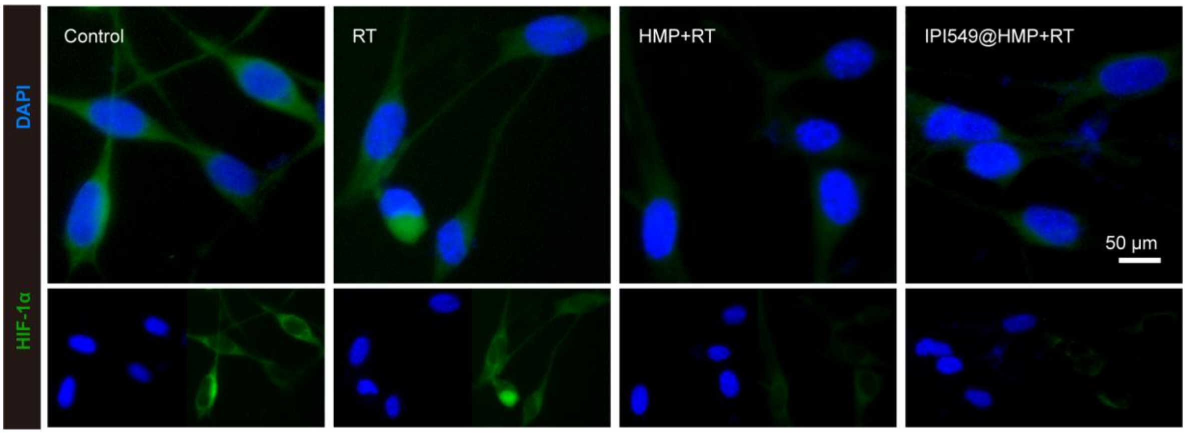

Supplementary Figure 12. HIF-1α expression within CT26 cells. Representative confocal images of CT26 tumor cells of three biologically independent samples from each group stained with HIF-1α antibody (green) and DAPI (blue) after varied treatments as indicated. RT, radiotherapy. The experiments were repeated three times.

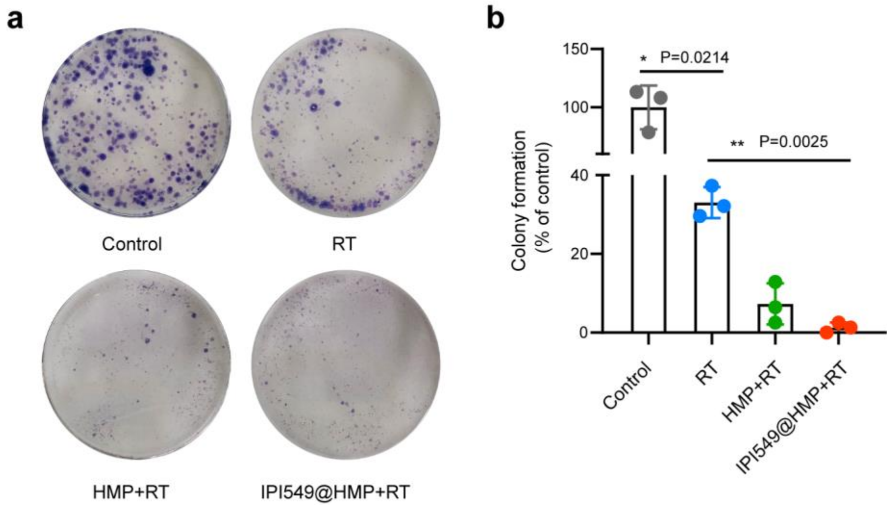

Supplementary Figure 13. colony formation assay. **a**, **b** Representative digital photo of three biologically independent samples from each group and corresponding quantitative analysis of CT26 cell colony formation after varied treatments. RT, radiotherapy. Data were expressed as mean ± SD (n = 3 independent samples). Statistical difference was calculated using two-tailed unpaired student's *t*-test. *P<0.05 and **P<0.01.

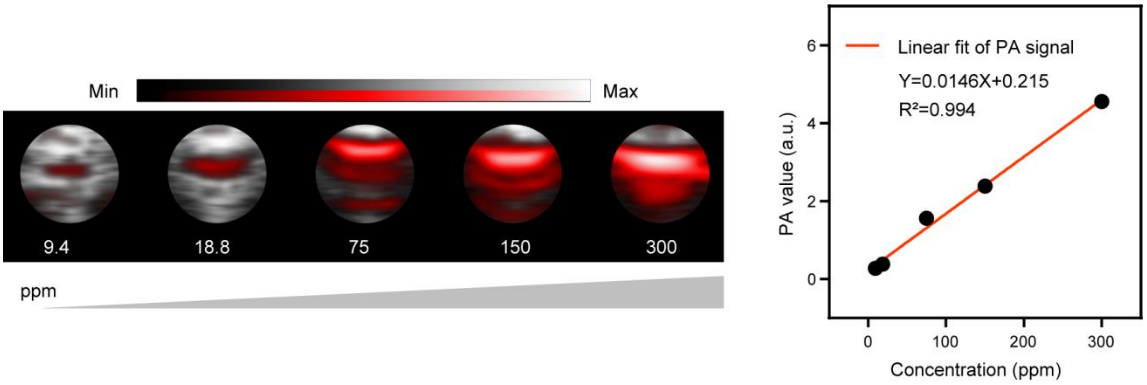

Supplementary Figure 14. Photoacoustic imaging capacity of HMP. Representative photoacoustic images and corresponding regression curve of HMP at varied concentrations (9.4, 18.8, 75, 150 and 300 ppm) from three independent samples.

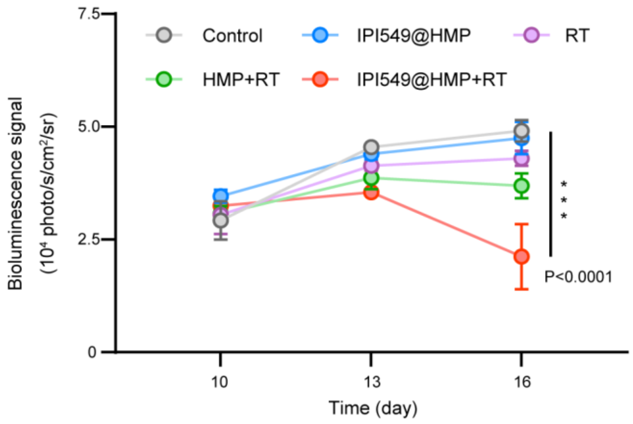

Supplementary Figure 15. The bioluminescence analysis of signals in control and treated groups. Data were expressed as means ± SD (n = 6 mice). RT, radiotherapy. Statistical difference was calculated using two-tailed unpaired student's *t*-test. ***P<0.001.

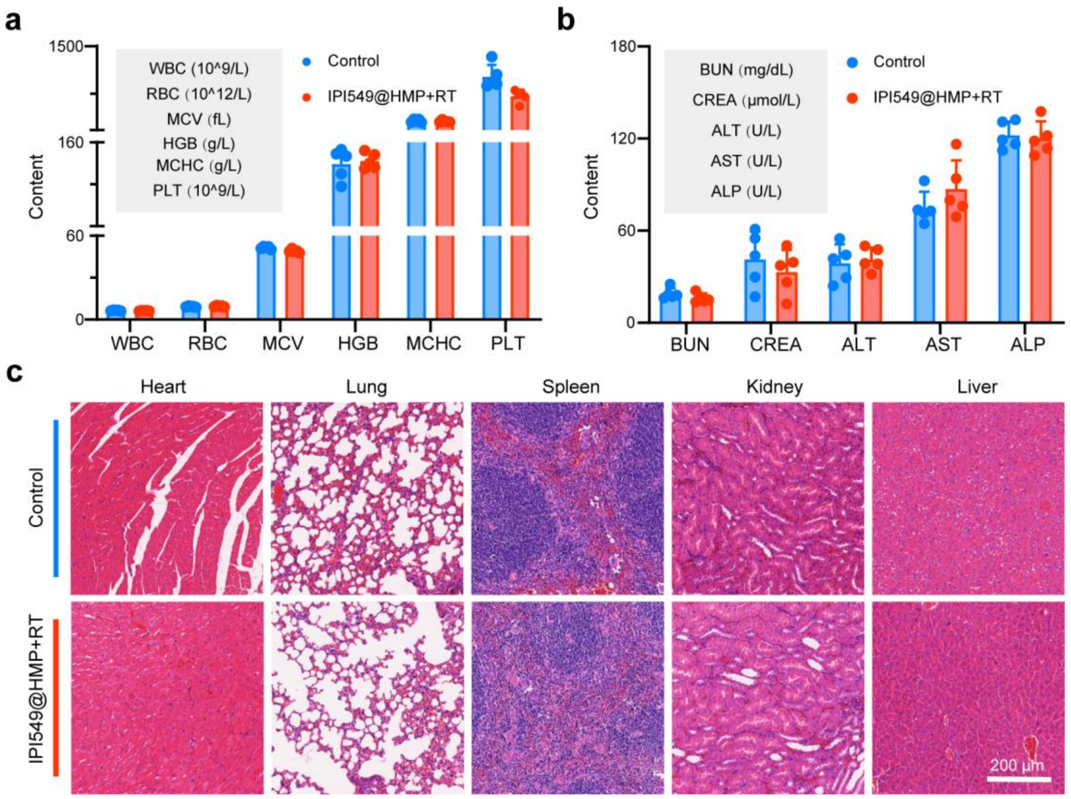

Supplementary Figure 16. *In vivo* safety evaluation after IPI549@HMP+RT treatment. **a** Blood routine indexes including leukocyte (WBC), red blood cell (RBC), mean corpuscular volume (MCV), hemoglobin (HGB), mean corpuscular hemoglobin concentration (MCHC) and platelet (PLT) were measured 7 days after varied treatment. Data were expressed as mean ± SD (n = 5 biologically independent animals). **b** Serum biochemistry data including blood urea nitrogen (BUN), creatinine (CREA), alanine aminotransferase (ALT), aspartate transaminase (AST) and alkaline phosphatase (ALP) were measured 7 days after varied treatment. Data were expressed as mean ± SD (n = 5 biologically independent animals). **c** Representative H&E stained tissue sections of three biologically independent animals from each group (All scale bar = 200 μm). RT, radiotherapy.

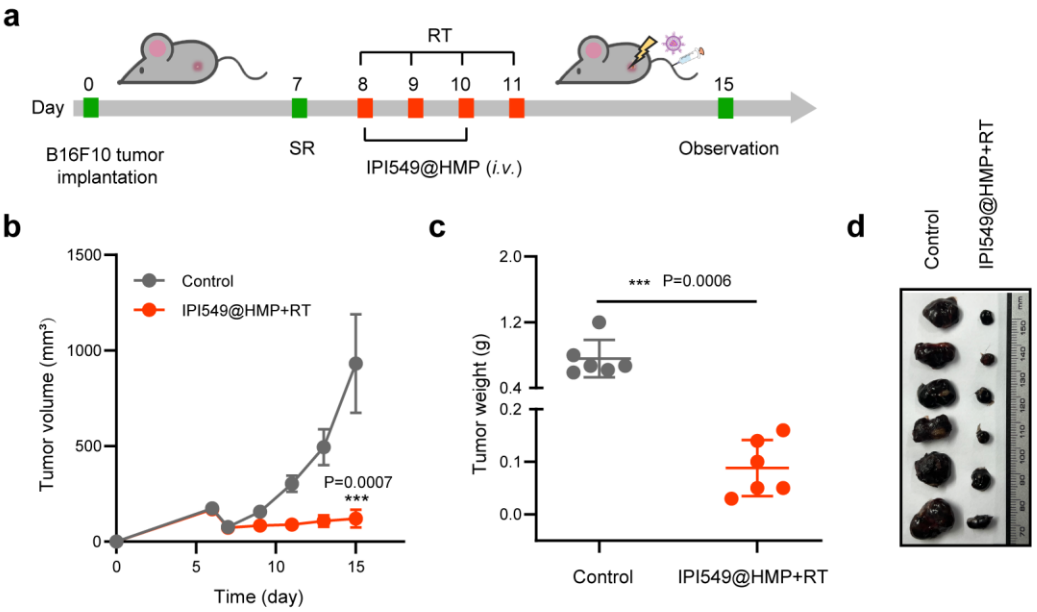

Supplementary Figure 17. IPI549@HMP-augmented RT against postsurgical melanoma. a Schematic illustration of the experiment design to assess the *in vivo* IPI549@HMP-based RT in B16F10 melanoma. **b** Residual tumor growth kinetics of mice after varied treatments. **c** Weight of the excised tumors examined on day 15 after varied treatments. **d** Digital photos of the excised tumors examined on day 15 after varied treatments. SR, surgical resection, RT, radiotherapy. Data were expressed as means ± SD (n = 6 mice in **b**-**d**). Statistical difference was calculated using two-tailed unpaired student's *t*-test. ***P<0.001.

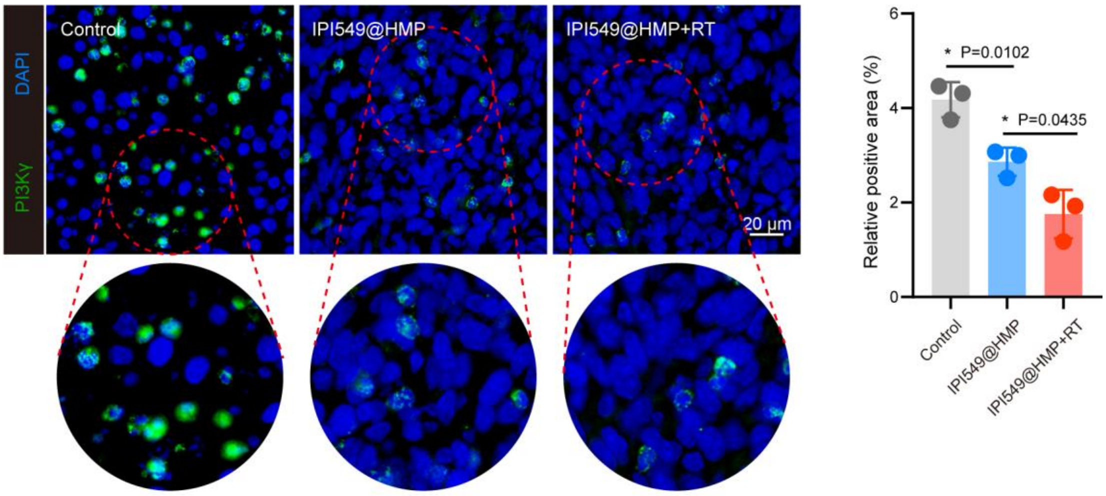

Supplementary Figure 18. PI3Kγ expression within tumor site after varied treatment. Immunofluorescence images and relative quantification of tumor slices stained with PI3Kγ antibody after varied treatments as indicated. Data were denoted as the mean ± SD (n = 3 biologically independent samples). Statistical difference was calculated using two-tailed unpaired student's *t*-test. *P<0.05. RT, radiotherapy.

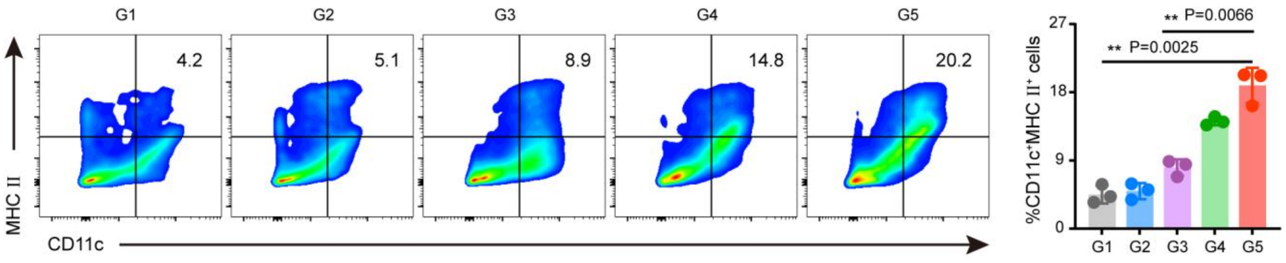

Supplementary Figure 19. Infiltration of dendritic cells in primary tumors. Representative flow cytometric images and relative quantification of dendritic cells (DCs, MHC II^+CD11c^+) within tumor tissues 9 days post treatment. G1: control; G2: IPI549@HMP; G3: RT; G4: HMP+RT; G5: IPI549@HMP+RT. Data were expressed as means ± SD (n = 3 biologically independent samples). Statistical difference was calculated using two-tailed unpaired student's *t*-test. **P<0.01.

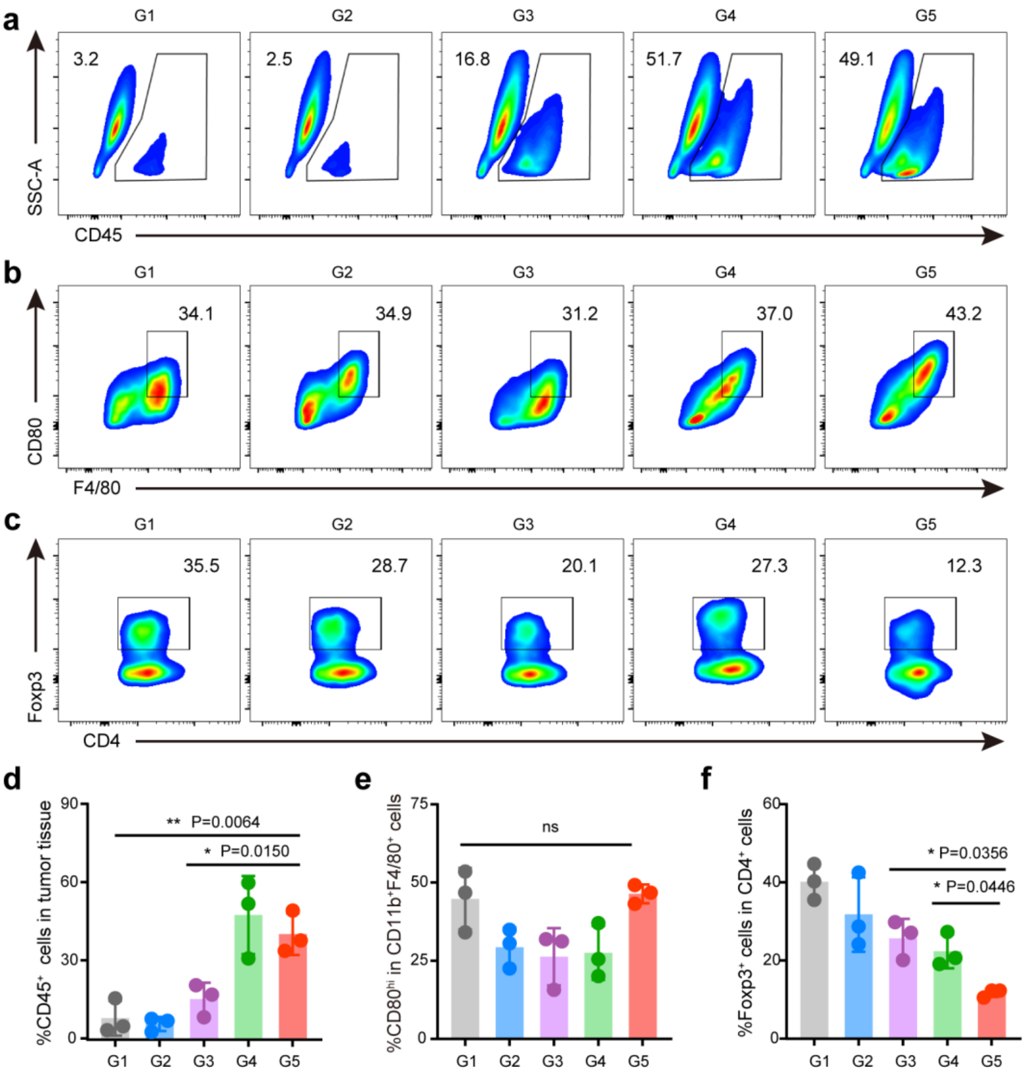

Supplementary Figure 20. Immune cells infiltration in primary tumors. a-f Representative flow cytometric images and relative quantification of CD45$^+$ cells (**a**, **d**), TAM-M1 (CD80^{hi}CD11b$^+$F4/80$^+$CD45$^+$) (**b**, **e**) and Treg cells (Foxp3$^+$CD4$^+$CD3$^+$CD45$^+$) (**c**, **f**) within tumor tissues 9 days post treatment. G1: control; G2: IPI549@HMP; G3: RT; G4: HMP+RT; G5: IPI549@HMP+RT. Data were expressed as means ± SD (n = 3 biologically independent samples in **d-f**). Statistical difference was calculated using two-tailed unpaired student's *t*-test. ns presents not significant, *P<0.05, **P<0.01.

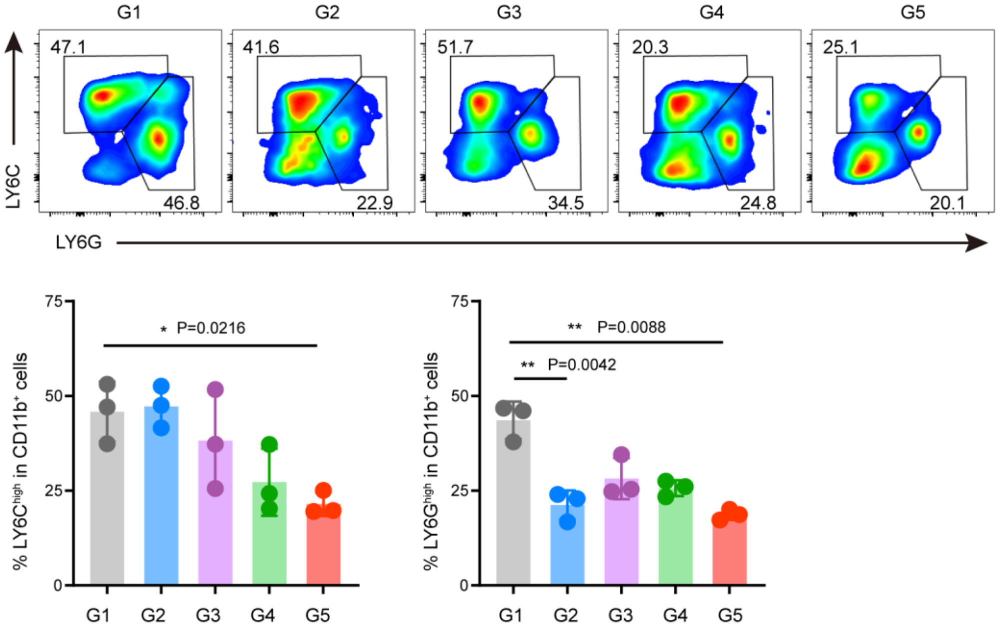

Supplementary Figure 21. Infiltration of MDSC subpopulations in primary tumors. Representative flow cytometric images and relative quantification of monocytes MDSC ($CD11b^{+}Ly6C^{high}$) and neutrophils MDSC ($CD11b^{+}Ly6G^{high}$) within tumor tissues 9 days post treatment. G1: control; G2: IPI549@HMP; G3: RT; G4: HMP+RT; G5: IPI549@HMP+RT. Data were expressed as means ± SD (n = 3 biologically independent samples). Statistical difference was calculated using two-tailed unpaired student's *t*-test. *P<0.05 and **P<0.01.

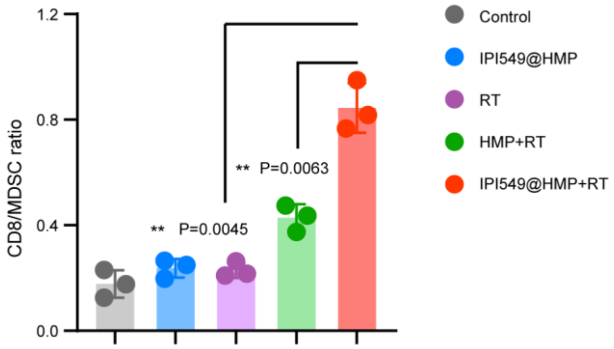

Supplementary Figure 22. Quantification by flow cytometry of CD8/MDSC ratios. RT, radiotherapy. Data were expressed as means ± SD (n = 3 biologically independent samples). Statistical difference was calculated using two-tailed unpaired student's *t*-test. **P<0.01.

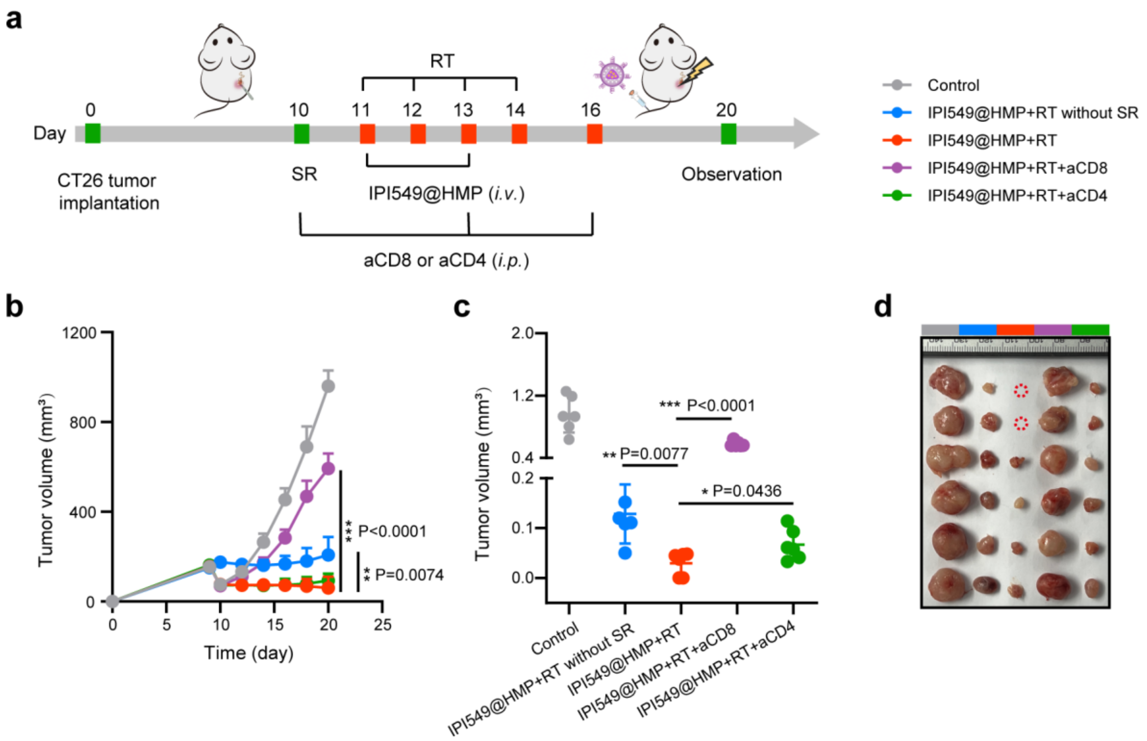

Supplementary Figure 23. a Schematic illustration of the experiment design to assess the *in vivo* IPI549@HMP-based RT in wild type CT26 tumors. **b** Average tumor growth kinetics of tumor-bearing mice after varied treatments. **c** Weight of the excised tumors examined on day 20 after varied treatments. **d** Digital photos of the excised tumors examined on day 20 after varied treatments. The circle represents cured tumors. SR, surgical resection, RT, radiotherapy. Data were expressed as means ± SD (n = 6 mice in **b**, **c** and **d**). Statistical difference was calculated using two-tailed unpaired student's *t*-test. *P<0.05, **P<0.01 and ***P<0.001.

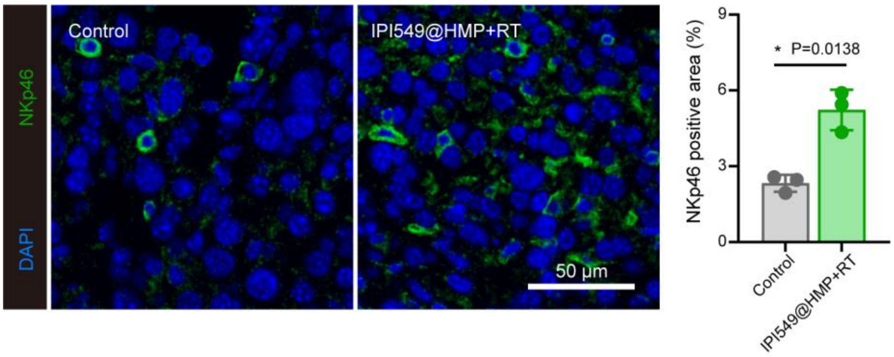

Supplementary Figure 24. NKp46 expression within primary tumor after varied treatment. Immunofluorescence images and relative quantification of tumor slices stained with NKp46 antibody after varied treatments as indicated. Data were denoted as the mean ± SD (n = 3 biologically independent samples). Statistical difference was calculated using two-tailed unpaired student's *t*-test. *P<0.05. RT, radiotherapy.

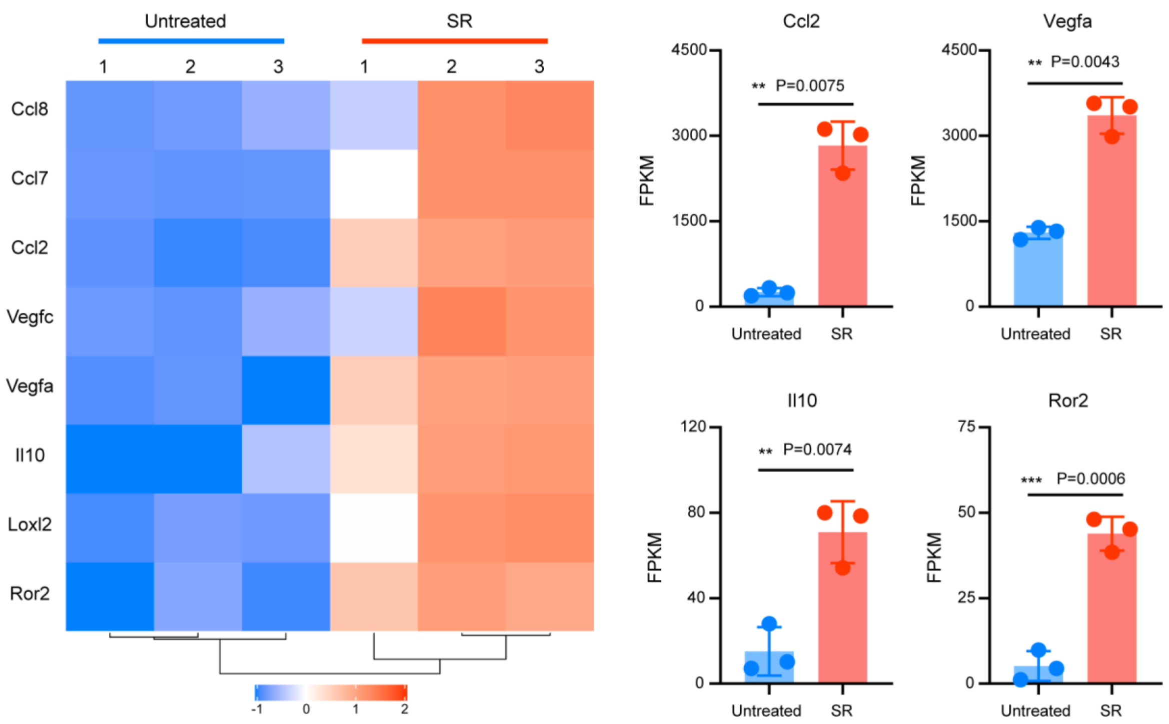

Supplementary Figure 25. SR up-regulates PD-L1-related genes expression. Heat map of differentially expressed PD-L1-related genes in untreated and SR-treated mice obtained by RNA sequencing. SR, surgical resection. Data were expressed as means ± SD (n = 3 biologically independent samples). Statistical difference was calculated using two-tailed unpaired student's *t*-test. **P<0.01, ***P<0.001.

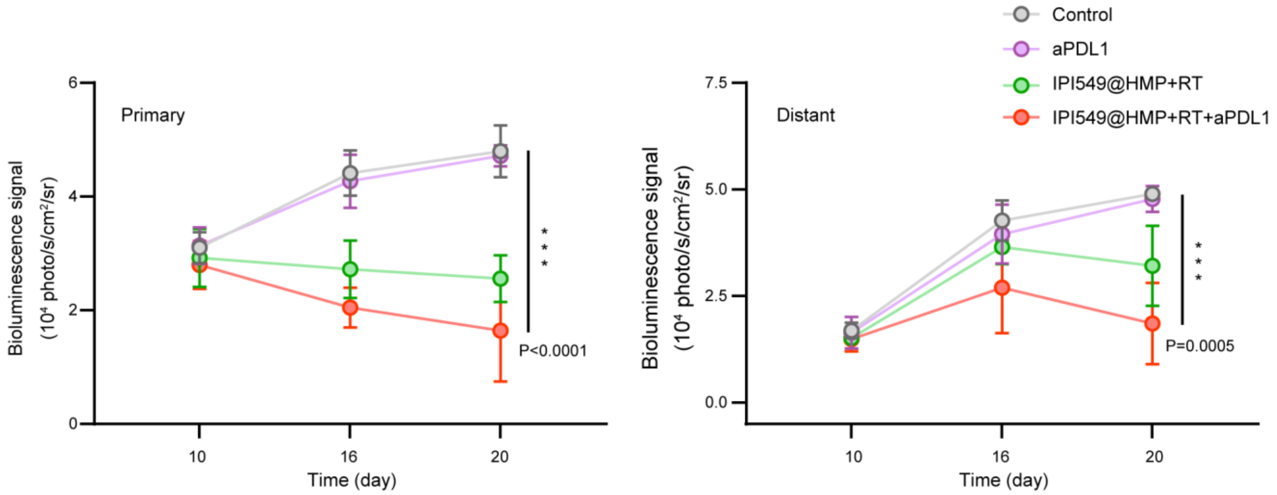

Supplementary Figure 26. The bioluminescence analysis of signals in control and treated groups. RT, radiotherapy. Data were expressed as means ± SD (n = 6 mice). Statistical difference was calculated using two-tailed unpaired student's *t*-test. ***P<0.001.

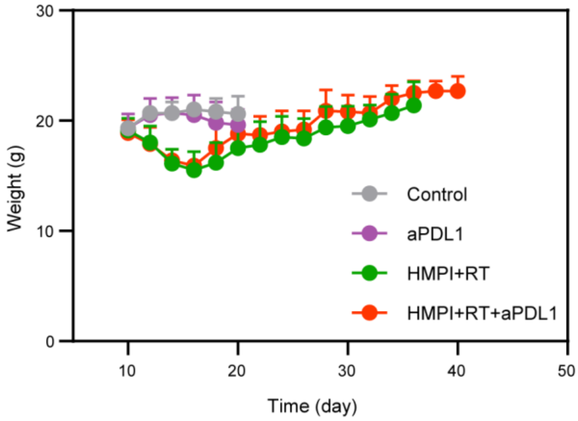

Supplementary Figure 27. Mice weight changes in bilateral model during observation. Body weight fluctuation curves of CT26 tumor-bearing mice treated with varied therapeutic combinations. RT, radiotherapy. Data were expressed as mean ± SD (n = 6 mice).

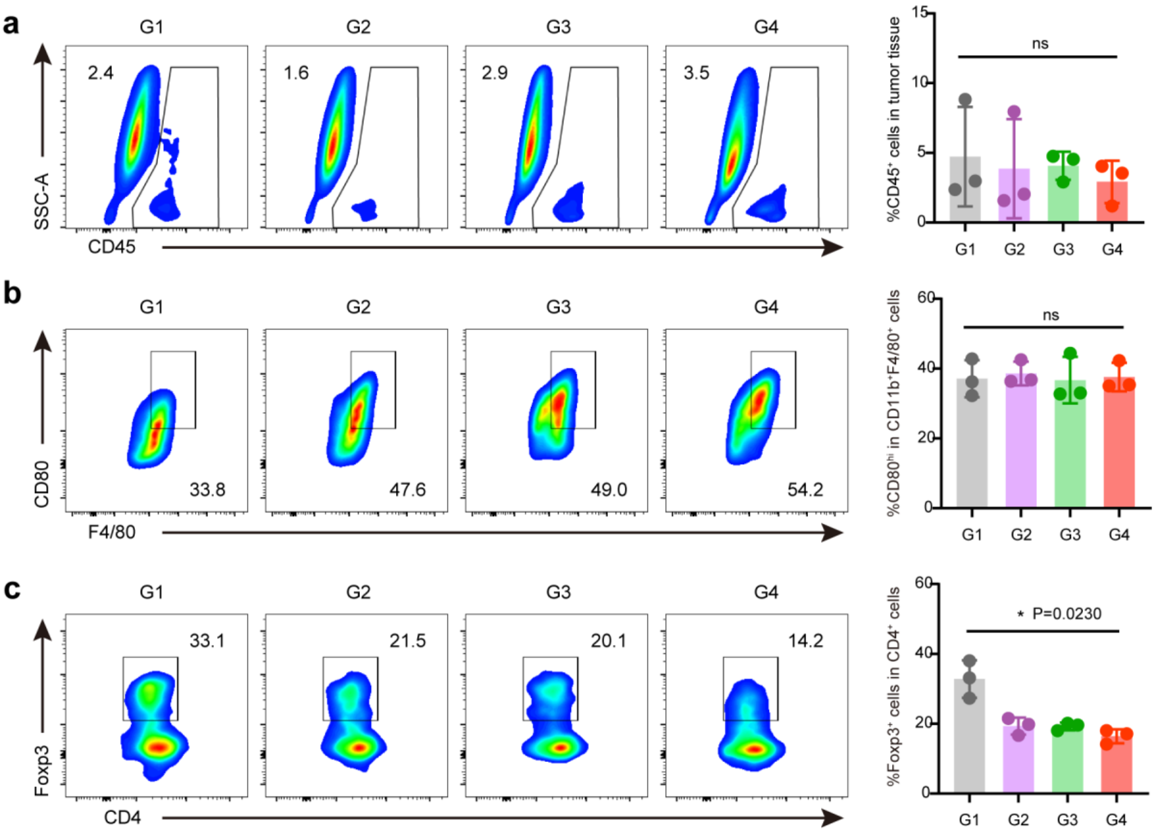

Supplementary Figure 28. Immune cells infiltration in distant tumors. a-c Representative flow cytometric images and relative quantification of CD45^{+} T cells (**a**), TAM-M1 (CD80^{hi}CD11b^{+}F4/80^{+}CD45^{+}) (**b**) and Treg cells (Foxp3^{+}CD4^{+}CD3^{+}CD45^{+}) (**c**) within tumor tissues 9 days after treatment. G1: control; G2: aPDL1; G3: IPI549@HMP+RT; G4: IPI549@HMP+RT+aPDL1. Data were expressed as means ± SD (n = 3 biologically independent samples in **a**, **b** and **c**). Statistical difference was calculated using two-tailed unpaired student's *t*-test. ns presents not significant, *P<0.05.

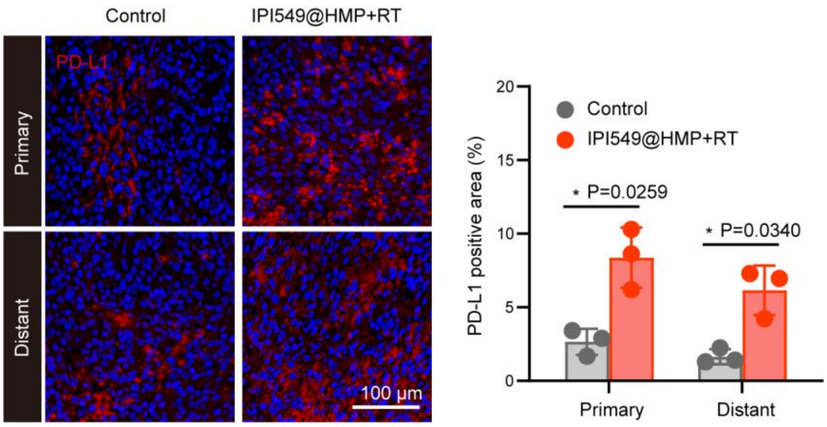

Supplementary Figure 29. PD-L1 expression in primary and distant tumors. Immunofluorescence and relative quantification of PD-L1 expression in mice before and 5 days after IPI549@HMP+RT treatment. Data were expressed as means ± SD (n = 3 biologically independent samples). Statistical difference was calculated using two-tailed unpaired student's t-test. *P<0.05. RT, radiotherapy.

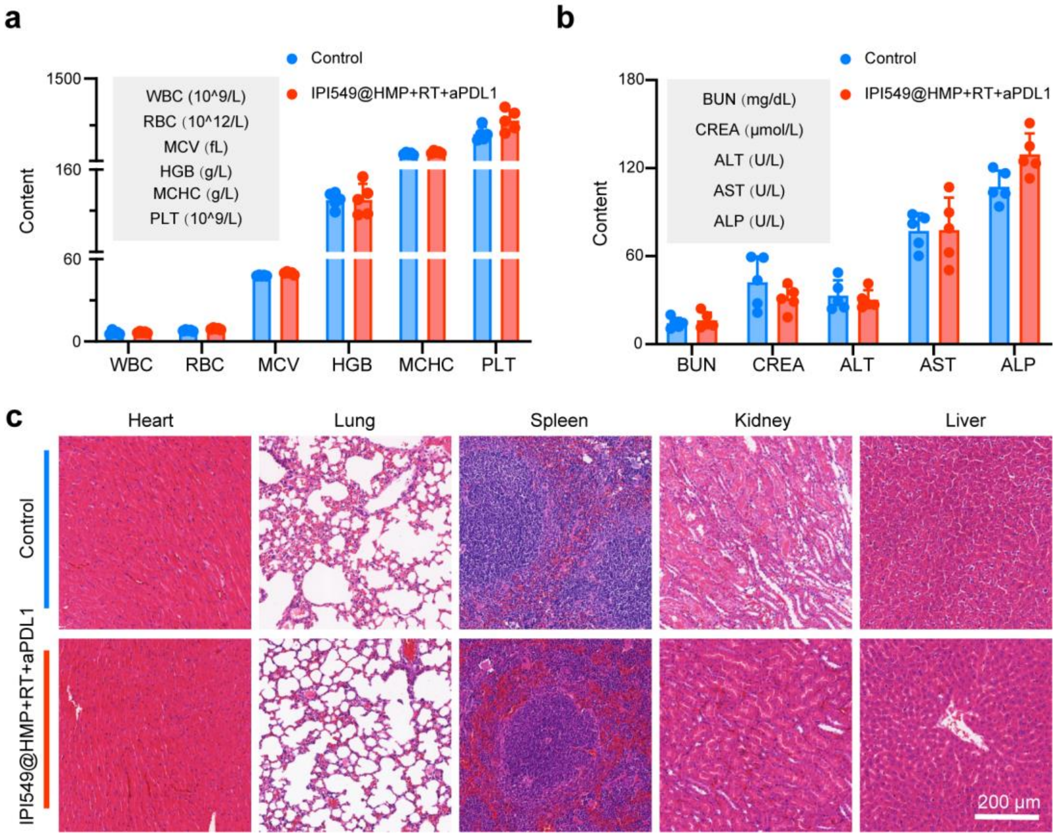

Supplementary Figure 30. ***In vivo* safety evaluation after IPI549@HMP+RT+aPDL1 treatment**. **a** Blood routine indexes including leukocyte (WBC), red blood cell (RBC), mean corpuscular volume (MCV), hemoglobin (HGB), mean corpuscular hemoglobin concentration (MCHC) and platelet (PLT) were measured 7 days after varied treatment. Data were expressed as mean ± SD (n = 5 biologically independent samples). **b** Serum biochemistry data including blood urea nitrogen (BUN), creatinine (CREA), alanine aminotransferase (ALT), aspartate transaminase (AST) and alkaline phosphatase (ALP) were measured 7 days after varied treatment. Data were expressed as mean ± SD (n = 5 biologically independent samples). **c** Representative H&E stained tissue sections of three biologically independent animals from each group (All scale bar = 200 μm). RT, radiotherapy.

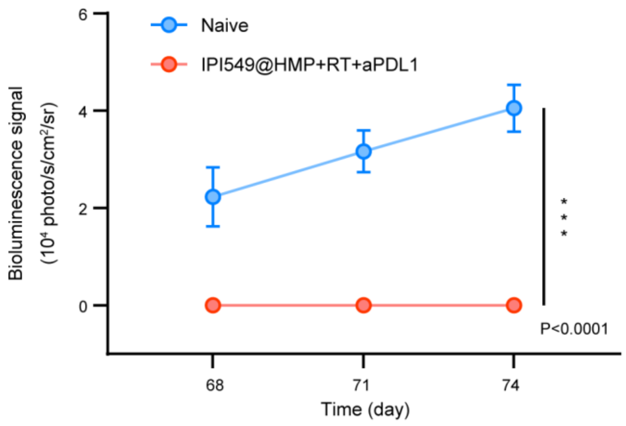

Supplementary Figure 31. The bioluminescence analysis of signals in naive and IPI549@HMP+RT+aPDL1-treated groups. RT, radiotherapy. Data were expressed as means ± SD (n = 6 mice). Statistical difference was calculated using two-tailed unpaired student's *t*-test. ***P<0.001.

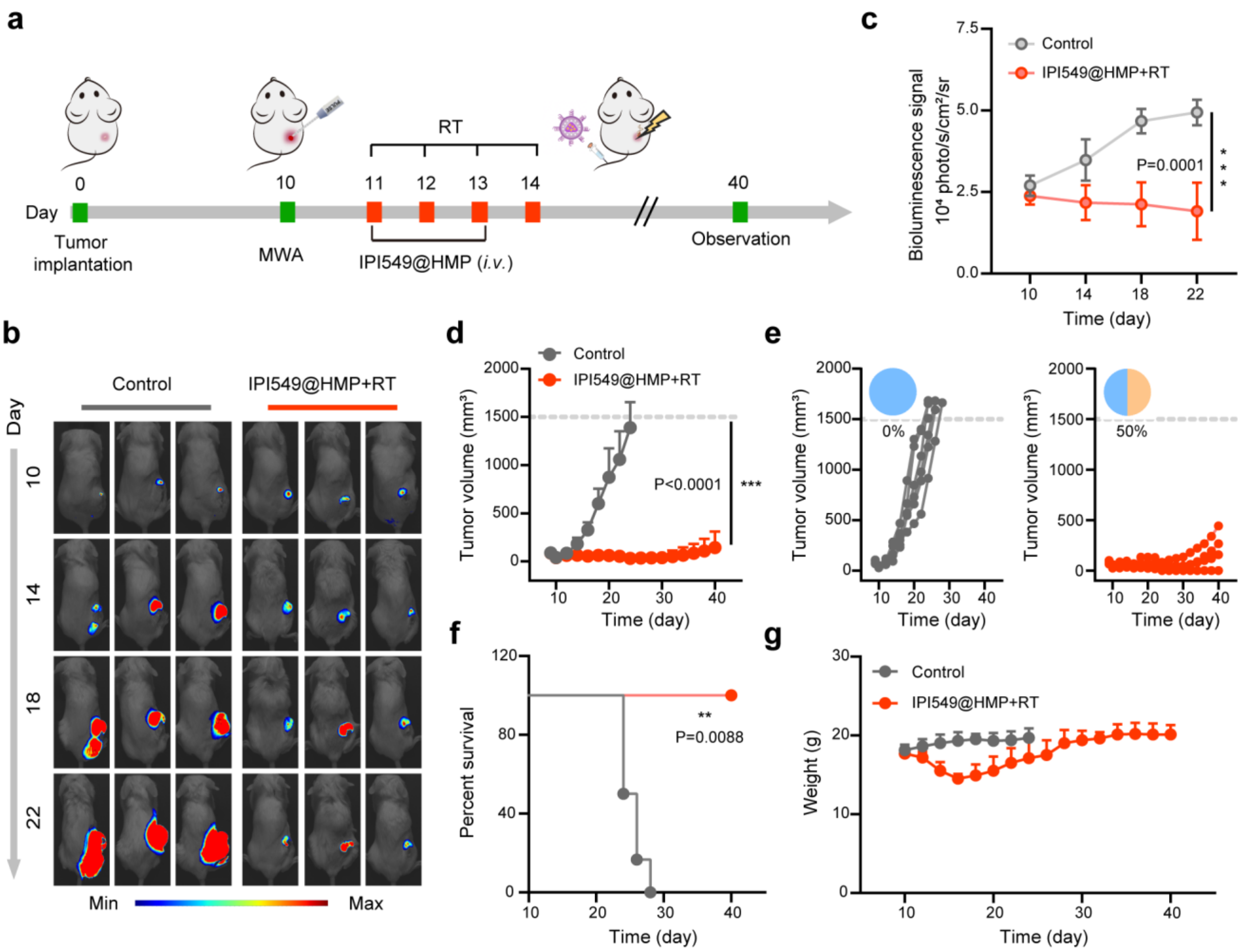

Supplementary Figure 32. IPI549@HMP-augmented RT against post-microwave ablation tumor progression. a Schematic illustration of the experiment design to assess the *in vivo* IPI549@HMP-based RT in Luc^+ CT26 tumors treated with ablation procedures. **b**, **c** Representative bioluminescence images of six biologically independent animals from each group (**b**) and relative quantification (**c**) of Luc^+ CT26 tumor taken on days 10, 14, 18 and 22 in control and IPI549@HMP+RT groups. **d-g** Average tumor growth curves (**d**), individual tumor growth kinetics (**e**), Kaplan-Meier survival curves (**f**) and body weight fluctuation curves (**g**) of Luc^+ CT26 tumor-bearing mice after varied therapeutic combinations. RT, radiotherapy. MWA, microwave ablation. Data were expressed as means ± SD (n = 6 mice in **c-g**). Statistical difference was calculated using two-tailed unpaired student's *t*-test (**c** and **d**) and Log-rank (Mantel-Cox) test (**f**). **P<0.01, ***P<0.001.

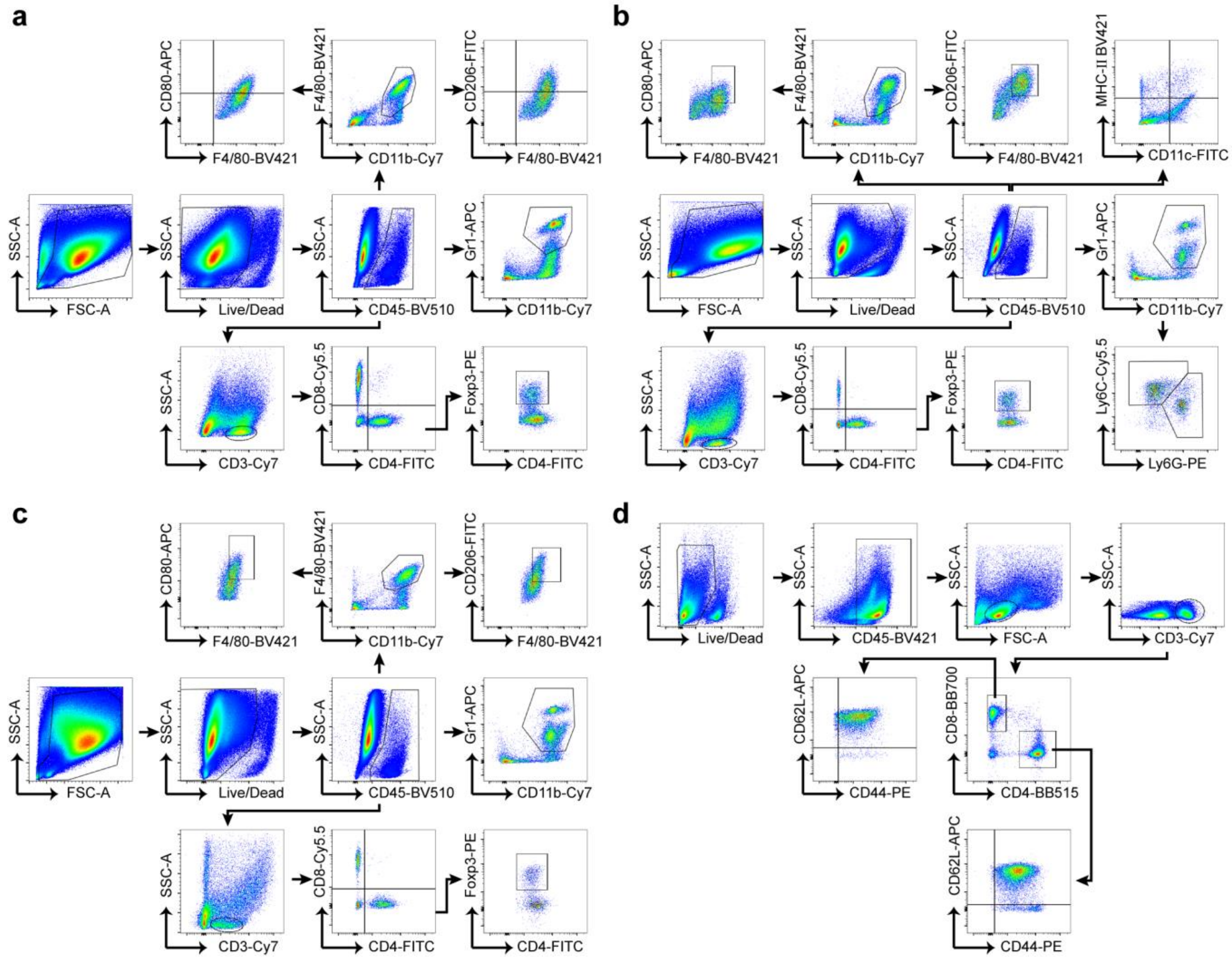

Supplementary Figure 33. Gating strategies for flow cytometry analysis of immune cells. a. Gating strategy to determine the percentage of MDSCs, TAMs and T cell subsets in $CD45^+$ immune cells, as displayed in Figure 2j-l and Supplementary Figure 3. **b** Gating strategy to determine the percentage of T cell subsets, MDSCs, TAMs and DCs in $CD45^+$ immune cells, as shown in Figure 6a, c, e and Supplementary Figure 19-21. **c** Gating strategy to determine the percentage of T cell subsets, MDSCs, TAMs in $CD45^+$ immune cells, as displayed in Figure 7f-h and Supplementary Figure 28. **d** Gating strategy to determine the percentage of Tcm and Tem subsets from $CD8^+$ and $CD4^+$ T cells in the spleen, as shown in Figure 8h. MDSCs, myeloid-derived suppressor cells; TAMs, tumor-associated macrophages; DCs, dendritic cells; Tcm, central memory T cell; Tem, effector memory T cell.
