# Supplementary material for: Nanoparticle-enhanced radiotherapy synergizes with PD-L1 blockade to limit post-surgical cancer recurrence and metastasis
Source: Nat Commun. 2022 May 20;13:2834. doi: 10.1038/s41467-022-30543-w (PMC9123179; doi:10.1038/s41467-022-30543-w)
Supplement: Supplementary file 2 — Reporting Summary [file 41467_2022_30543_MOESM2_ESM.pdf]

nature portfolio

Corresponding author(s): Yu Chen; Huixiong Xu; Wenen Yue

Last updated by author(s): Apr 16, 2022

Reporting Summary

Nature Portfolio wishes to improve the reproducibility of the work that we publish. This form provides structure for consistency and transparency in reporting. For further information on Nature Portfolio policies, see our Editorial Policies and the Editorial Policy Checklist.

Statistics

For all statistical analyses, confirm that the following items are present in the figure legend, table legend, main text, or Methods section.

n/a	Confirmed	
☐	☒	The exact sample size (n) for each experimental group/condition, given as a discrete number and unit of measurement
☐	☒	A statement on whether measurements were taken from distinct samples or whether the same sample was measured repeatedly
☐	☒	The statistical test(s) used AND whether they are one- or two-sided<br>*Only common tests should be described solely by name; describe more complex techniques in the Methods section.*
☐	☒	A description of all covariates tested
☐	☒	A description of any assumptions or corrections, such as tests of normality and adjustment for multiple comparisons
☐	☒	A full description of the statistical parameters including central tendency (e.g. means) or other basic estimates (e.g. regression coefficient) AND variation (e.g. standard deviation) or associated estimates of uncertainty (e.g. confidence intervals)
☐	☒	For null hypothesis testing, the test statistic (e.g. F, t, r) with confidence intervals, effect sizes, degrees of freedom and P value noted<br>*Give P values as exact values whenever suitable.*
☒	☐	For Bayesian analysis, information on the choice of priors and Markov chain Monte Carlo settings
☒	☐	For hierarchical and complex designs, identification of the appropriate level for tests and full reporting of outcomes
☒	☐	Estimates of effect sizes (e.g. Cohen's d, Pearson's r), indicating how they were calculated

Our web collection on statistics for biologists contains articles on many of the points above.

Software and code

Policy information about availability of computer code

Data collection	TEM (FEI Tecnai G2 F30), laser granularity sizer (Zetasizer Nano ZS90), UV-vis-NIR spectrophotometer (PE Lambda 950), Accelerated Surface Area and Porosimetry System (ASAP 2460), X-ray photo-electron spectroscopy (XPS, Thermo escalab 250Xi), Nikon bio-microscope (CI-L), Inductively coupled plasma mass spectrometry (ICP-MS, Agilent ICPMS7800), 3.0 T MR clinical scanner (United Imaging, uMR 770), portable dissolved oxygen analyzer (Leici JPB-607A), Photoacoustic imaging system (VEVO LAZR-X), FACS flow cytometry (BD, Canto ?), fluorescent scanning camera (KFBIO, KF-TB-400).
Data analysis	All statistical analyses were performed on Graphpad Prism (version 8). All flowcytometry data were analyzed on FlowJo software package (TreeStar, version 10.6.2). Living imaging software (VISQUE In vivo Smart-LF, version 3.1.3.2054) was used to analyse bioluminescent and fluorescent images. All immunohistochemistry and immunofluorescence images were quantified with Image J (version 1.8.0).Magnetic resonance data were processed with an MR system (uExeed, R002).

For manuscripts utilizing custom algorithms or software that are central to the research but not yet described in published literature, software must be made available to editors and reviewers. We strongly encourage code deposition in a community repository (e.g. GitHub). See the Nature Portfolio guidelines for submitting code & software for further information.

Data

Policy information about availability of data

All manuscripts must include a data availability statement. This statement should provide the following information, where applicable:

- Accession codes, unique identifiers, or web links for publicly available datasets
- A description of any restrictions on data availability
- For clinical datasets or third party data, please ensure that the statement adheres to our policy

The raw sequencing data generated in this study have been deposited in the Genome Sequence Archive (GSA) database under accession code CRA006269. The remaining data are available within the Article, Supplementary Information or Source Data file. Source data are provided with this paper.

Field-specific reporting

Please select the one below that is the best fit for your research. If you are not sure, read the appropriate sections before making your selection.

☒ Life sciences ☐ Behavioural & social sciences ☐ Ecological, evolutionary & environmental sciences

For a reference copy of the document with all sections, see nature.com/documents/nr-reporting-summary-flat.pdf

Life sciences study design

All studies must disclose on these points even when the disclosure is negative.

Sample size	Although no sample size calculation was performed, sample sizes (at least three animals per treatment group) represents the minimum number animals needed to reach statistical significance ($p < 0.05$) between experimental groups. Meanwhile, sample sizes for the in vivo experiments are similar to those generally employed and accepted in the field (Nat Commun 12, 3187 (2021); Nat Nanotechnol 16, 1271-1280 (2021)) and were sufficient to support our conclusions with statistical significance. Sample sizes for the in vitro experiments are also based on previous work (Nat Nanotechnol 16, 538-548 (2021)).
Data exclusions	No data were excluded.
Replication	Experiment were repeated and experimental findings were reproducible. Details of experimental replicates are given in the figure legends.
Randomization	All experimental samples or models were allocated randomly to each group.
Blinding	The investigator were not blinded for most of experiments since the experimental design, execution and data analysis were performed by the same person. Bioluminescence imaging were conducted by an independent operator, who was unaware of the treatment conditions.

Reporting for specific materials, systems and methods

We require information from authors about some types of materials, experimental systems and methods used in many studies. Here, indicate whether each material, system or method listed is relevant to your study. If you are not sure if a list item applies to your research, read the appropriate section before selecting a response.

Materials & experimental systems

n/a	Involved in the study	
☐	☒	Antibodies
☐	☒	Eukaryotic cell lines
☒	☐	Palaeontology and archaeology
☐	☒	Animals and other organisms
☒	☐	Human research participants
☒	☐	Clinical data
☒	☐	Dual use research of concern

Methods

n/a	Involved in the study	
☒	☐	ChIP-seq
☐	☒	Flow cytometry
☒	☐	MRI-based neuroimaging

Antibodies

Antibodies used	The following primary antibodies were used for immunofluorescence. They are listed as antigen first, followed by supplier, catalog number and clone number as applicable. All the antibodies were diluted and used following the supplier protocol.<br>1) Anti-mouse CD45 (abcam, Catalog No.ab10558)<br>2) Anti-mouse CD11b (abcam, Catalog No.ab133357, Clone No. EPR1344)<br>3) Ki67 (abcam, Catalog No.ab15580)<br>4) HIF-1α (Bioss, Catalog No.bs-0737R)<br>5) Anti-mouse CD206 (CST, Catalog No.24595, Clone No. E6T5J)

6) Anti-mouse CD8 (CST, Catalog No.98941, Clone No. D4W2Z)
7) Anti-mouse Foxp3 (abcam, Catalog No.ab215206, Clone No. EPR22102-37)
8) γ-H2AX antibody (CST, Catalog No.80312S, Clone No. D7T2V)
9) HMGB1 antibody(CST, Catalog No.3935)
10) calreticulin (CST, Catalog No.12238, Clone No. D3E6)
11) anti-NKp46 (Abcam, Catalog No. ab233558, Clone No. EPR23097-35)
12) anti-PD-L1 (CST, Catalog No. 64988, Clone No. D5V3B)
The following primary antibodies were used for western blotting. They are listed as antigen first, followed by supplier, catalog number and clone number as applicable. All the antibodies were diluted and used following the supplier protocol.
1) anti-PI3K p110 gamma antibody (CST, Catalog No.5405T, Clone No. D55D5)
2) anti-GADPH antibody (CST, Catalog No.5174T, Clone No. D16H11)
3) anti-rabbit lgG-HRP (CST, Catalog No.5127S, Clone No. L27A9)
The following primary antibodies were used for flow cytometry. They are listed as antigen first, followed by supplier, catalog number and clone number as applicable. All the antibodies were diluted and used following the supplier protocol.
1) CD16/CD32 (eBioscience, Catalog No. MFCR00, Clone No. FRC-4G8)
2) CD45-eF506 (eBioscience, Catalog No.69-0451-82, Clone No. 30-F11)
3) CD3-PE-cy7 (eBioscience, Catalog No.25-0031-82, Clone No. 145-2C11)
4) CD4-FITC (eBioscience, Catalog No.11-0041-82, Clone No. GK1.5)
5) CD8-Percp-cy5.5 (eBioscience, Catalog No.45-0081-82, Clone No. 53-6.7)
6) Foxp3-PE (eBioscience, Catalog No.12-5773-82, Clone No. FJK-16s)
7) CD11b-PE-cy7 (eBioscience, Catalog No.25-0112-82, Clone No. M1/70)
8) CD11c-PE (eBioscience, Catalog No.12-0114-82, Clone No. N418)
9) MHC-II-eF450 (eBioscience, Catalog No.48-5321-82, Clone No. M5/114.15.2)
10) Ly6c-percp-Cy5.5 (eBioscience, Catalog No.45-5932-82, Clone No. HK1.4)
11) Ly6G-PE (eBioscience, Catalog No.12-9668-82, Clone No. 1A8-Ly6g)
12) Gr-1-APC (Biolegend, Catalog No.108412, Clone No. RB6-8C5)
13) F4/80-BV421 (Biolegend, Catalog No.123137, Clone No. BM8)
14) CD206-FITC (Biolegend, Catalog No.141703, Clone No. C068C2)
15) CD80-APC (Biolegend, Catalog No.104714, Clone No. 16-10A1)
16) CD62L-APC (eBioscience, Catalog No. 17-0621, Clone No. MEL-14)
17) CD44-PE (eBioscience, Catalog No. 12-0441, Clone No. IM7)
The following antibodies were used for in vivo depletion experiments. They are listed as antigen first, followed by supplier, catalog number and clone number as applicable. All the antibodies were diluted and used following the supplier protocol.
1) In vivo anti-mouse CD8 (BioXell, Catalog No.BE0004, Clone No. 53-6.7)
2) In vivo anti-mouse CD4 (BioXell, Catalog No.BE0003, Clone No. GK1.5)
3) In vivo anti-mouse PDL1 (BioXell, Catalog No.BE0101, Clone No. B7-H1)

Validation

All antibodies used in this manuscript were commercially available. The validation and quality control are performed by the corresponding vendors, and available on the manufactures' website and datasheet:
The following primary antibodies were used for immunofluorescence
1) Anti-mouse CD45: https://www.abcam.cn/cd45-antibody-ab10558.html
2) Anti-mouse CD11b: https://www.abcam.cn/cd11b-antibody-epr1344-ab133357.html
3) Ki67: https://www.abcam.cn/ki67-antibody-ab15580.html
4) HIF-1α: http://www.bioss.com.cn/prolook_03.asp?id=AF08169606000431&pro37=1
5) Anti-mouse CD206: https://www.cellsignal.cn/products/primary-antibodies/cd206-mrc1-e6t5j-xp-rabbit-mab/24595?site-search-type=Products&N=4294956287&Ntt=24595&fromPage=plp&_requestid=4408348
6) Anti-mouse CD8: https://www.cellsignal.cn/products/primary-antibodies/cd8a-d4w2z-xp-rabbit-mab-mouse-specific/98941?site-search-type=Products&N=4294956287&Ntt=98941&fromPage=plp&_requestid=4408393
7) Anti-mouse Foxp3: https://www.abcam.cn/foxp3-antibody-epr22102-37-ab215206.html
8) γ-H2AX antibody: https://www.cellsignal.cn/products/primary-antibodies/phospho-histone-h2a-x-ser139-d7t2v-mouse-mab/80312?site-search-type=Products&N=4294956287&Ntt=80312s&fromPage=plp&_requestid=4408441
9) HMGB1 antibody: https://www.cellsignal.cn/products/primary-antibodies/hmgb1-antibody/3935?site-search-type=Products&N=4294956287&Ntt=3935&fromPage=plp&_requestid=4408533
10) calreticulin: https://www.cellsignal.cn/products/primary-antibodies/calreticulin-d3e6-xp-rabbit-mab/12238?_=1649916067530&Ntt=12238&tahead=true
11) anti-NKp46: https://www.abcam.cn/ncr1-antibody-epr23097-35-ab233558.html
12) anti-PD-L1: https://www.cellsignal.cn/products/primary-antibodies/pd-l1-d5v3b-rabbit-mab-mouse-specific-ihc-specific/64988?site-search-type=Products&N=4294956287&Ntt=64988&fromPage=plp&_requestid=4408611
The following primary antibodies were used for western blotting
1) anti-PI3K p110 gamma antibody: https://www.cellsignal.cn/products/primary-antibodies/pi3-kinase-p110g-d55d5-rabbit-mab/5405;jsessionid=w064fn2nsf9lcwcrebber33mt_jdevyi4lo2kvv_.prod_store04?N=4294956287&Ntt=5405t&_requestid=4394409&fromPage=plp&site-search-type=Products
2) anti-GADPH antibody: https://www.cellsignal.cn/products/primary-antibodies/gapdh-d16h11-xp-rabbit-mab/5174;jsessionid=y6qqeblz6uyp4rmqbetzhqvolzyhchn-tgrakhsw.prod_store02?N=4294956287&Ntt=5174t&_requestid=4387291&fromPage=plp&site-search-type=Products
3) anti-rabbit lgG-HRP: https://www.cellsignal.cn/products/secondary-antibodies/mouse-anti-rabbit-igg-conformation-specific-l27a9-mab-hrp-conjugate/5127;jsessionid=ku6ucdrwqu8eaqezaedq1cqupr-lpemamsqadeic.prod_store01?N=4294956287&Ntt=5127s&_requestid=4440384&fromPage=plp&site-search-type=Products
The following primary antibodies were used for flow cytometry. They are listed as antigen first, followed by supplier, catalog number and clone number as applicable. All the antibodies were diluted and used following the supplier protocol.
1) CD16/CD32: https://www.thermofisher.cn/cn/zh/antibody/product/CD16-CD32-Antibody-clone-FRC-4G8-Monoclonal/MFCR00
2) CD45-eF506: https://www.thermofisher.cn/cn/zh/antibody/product/CD45-Antibody-clone-30-F11-Monoclonal/69-0451-82
3) CD3-PE-cy7: https://www.thermofisher.cn/cn/zh/antibody/product/CD3e-Antibody-clone-145-2C11-Monoclonal/25-0031-82
4) CD4-FITC: https://www.thermofisher.cn/cn/zh/antibody/product/CD4-Antibody-clone-GK1-5-Monoclonal/11-0041-82
5) CD8-Percp-cy5.5: https://www.thermofisher.cn/cn/zh/antibody/product/CD8a-Antibody-clone-53-6-7-Monoclonal/45-0081-82
6) Foxp3-PE: https://www.thermofisher.cn/cn/zh/antibody/product/FOXP3-Antibody-clone-FJK-16s-Monoclonal/12-5773-82

7) CD11b-PE-cy7: https://www.thermofisher.cn/cn/zh/antibody/product/CD11b-Antibody-clone-M1-70-Monoclonal/25-0112-82
8) CD11c-PE: https://www.thermofisher.cn/cn/zh/antibody/product/CD11c-Antibody-clone-N418-Monoclonal/12-0114-82
9) MHC-II-eF450: https://www.thermofisher.cn/cn/zh/antibody/product/MHC-Class-II-I-A-I-E-Antibody-clone-M5-114-15-2-Monoclonal/48-5321-82
10) Ly6c-percp-Cy5.5: https://www.thermofisher.cn/cn/zh/antibody/product/Ly-6C-Antibody-clone-HK1-4-Monoclonal/45-5932-82
11) Ly6G-PE: https://www.thermofisher.cn/cn/zh/antibody/product/Ly-6G-Antibody-clone-1A8-Ly6g-Monoclonal/12-9668-82
12) Gr-1-APC: https://www.biolegend.com/en-us/products/apc-anti-mouse-ly-6g-ly-6c-gr-1-antibody-456
13) F4/80-BV421: https://www.biolegend.com/en-us/products/brilliant-violet-421-anti-mouse-f4-80-antibody-7199
14) CD206-FITC: https://www.biolegend.com/en-us/products/fitc-anti-mouse-cd206-mmr-antibody-7318
15) CD80-APC: https://www.biolegend.com/en-us/products/apc-anti-mouse-cd80-antibody-2340
16) CD62L-APC: https://www.thermofisher.cn/cn/zh/antibody/product/CD62L-L-Selectin-Antibody-clone-MEL-14-Monoclonal/17-0621-81
17) CD44-PE: https://www.thermofisher.cn/cn/zh/antibody/product/CD44-Antibody-clone-IM7-Monoclonal/12-0441-82
The following antibodies were used for in vivo depletion experiments. They are listed as antigen first, followed by supplier, catalog number and clone number as applicable. All the antibodies were diluted and used following the supplier protocol.
1) In vivo anti-mouse CD8: https://bxcell.com/product/m-cd8a/
2) In vivo anti-mouse CD4: https://bxcell.com/product/m-cd4-3/
3) In vivo anti-mouse PDL1: https://bxcell.com/product/m-pdl-1/

Eukaryotic cell lines

Policy information about cell lines

Cell line source(s)	Mouse CT26 cells and B16F10 cells were originally obtained from ATCC. Luc+CT26 cells were obtained from Shanghai Zhong Qiao Xin Zhou Biotechnology Co. Ltd.
Authentication	Identity of the cell lines were frequently checked by their morphological features.
Mycoplasma contamination	All cell lines were tested for mycoplasma contamination. No mycoplasma contamination was found.
Commonly misidentified lines (See ICLAC register)	No commonly misidentified cell lines were used.

Animals and other organisms

Policy information about studies involving animals; ARRIVE guidelines recommended for reporting animal research

Laboratory animals	Female BALB/c mice (6-8 weeks) and female C57BL/6 mice (6-8 weeks) were purchased from Shanghai Salccas Biotechnology Co., Ltd. All mice were housed in an SPF-grade pathogen-free facility with a 12 h light/dark cycle at 20 ± 3? and a relative humidity of 40% to 70%.
Wild animals	The study did not involve wild animals.
Field-collected samples	The study did not involve samples collected from field.
Ethics oversight	All animal experiments were under the context of the animal protocols approved by the Institutional Animal Care and Use Committee guidelines in Shanghai Tenth Peoples' Hospital. All mice were kept in accordance with the policies on animal research of the National Ministry of Health.

Note that full information on the approval of the study protocol must also be provided in the manuscript.

Flow Cytometry

Plots

Confirm that:

☒ The axis labels state the marker and fluorochrome used (e.g. CD4-FITC).

☒ The axis scales are clearly visible. Include numbers along axes only for bottom left plot of group (a 'group' is an analysis of identical markers).

☒ All plots are contour plots with outliers or pseudocolor plots.

☒ A numerical value for number of cells or percentage (with statistics) is provided.

Methodology

Sample preparation	For all samples, cells were first stained with antibodies against surface antigens. In some experiments, cells were subsequently fixed, permeablized and stained for intracellular antigens. For tissue sample, the tissue was first mechanically disrupted from mice and divided into small pieces and homogenized in cold staining buffer to form single cell suspensions in the presence of digestive enzyme.
Instrument	BD Fortessa X20

Software	FlowJo software package (version 10.6.2).
Cell population abundance	No sorting was performed.
Gating strategy	Generally, cells were first gated on FSC/SSC. Singlet cells were gated using FSC-H and FSC-A. Surface antigen gating was performed on the live cell population.

☒ Tick this box to confirm that a figure exemplifying the gating strategy is provided in the Supplementary Information.
